# Uncovering specific changes in network wiring underlying the primate cerebrotype

**DOI:** 10.1007/s00429-017-1402-6

**Published:** 2017-03-25

**Authors:** Salah Hamodeh, Ayse Bozkurt, Haian Mao, Fahad Sultan

**Affiliations:** 1 Department of Cognitive Neurology, HIH for Clinical Brain Research, Otfried-Müller-Str. 27, 72076 Tübingen, Germany; 2 Department of Integrative Medical Biology, Umeå University, Linnéus väg 9, 901 87 Umeå, Sweden

**Keywords:** Cerebellar nuclei, Dentate nucleus, Motor systems, Purkinje cells, Quantitative immunohistochemistry, 3D reconstructions, Comparative neuroanatomy

## Abstract

Regular scaling of brain networks during evolution has been proposed to be the major process leading to enlarged brains. Alternative views, however, suggest that deviations from regular scaling were crucial to the evolution of the primate brain and the emergence of different cerebrotypes. Here, we examined the scaling within the major link between the cerebellum and the cerebral cortex by studying the deep cerebellar nuclei (DCN). We compared the major axonal and dendritic wiring in the DCN of rodents and monkeys in search of regular scaling. We were able to confirm regular scaling within the density of neurons, the general dendritic length per neuron and the Purkinje cell axon length. However, we also observed specific modification of the scaling rules within the primates’ largest and phylogenetically newest DCN, the dentate nucleus (LN/dentate). Our analysis shows a deviation from regular scaling in the predicted dendritic length per neuron in the LN/dentate. This reduction in the dendritic length is also associated with a smaller dendritic region-of-influence of these neurons. We also detected specific changes in the dendritic diameter distribution, supporting the theory that there is a shift in the neuronal population of the LN/dentate towards neurons that exhibit spatially restricted, clustered branching trees. The smaller dendritic fields would enable a larger number of network modules to be accommodated in the primate LN/dentate and would provide an explanation for the unique folded structure of the primate LN/dentate. Our results show that, in some brain regions, connectivity maximization (i.e., an increase of dendritic fields) is not the sole optimum and that increases in the number of network modules may be important for the emergence of a divergent primate cerebrotype.

**Electronic supplementary material:**

The online version of this article (doi:10.1007/s00429-017-1402-6) contains supplementary material, which is available to authorized users.

## Introduction

Brains of different sizes can show a remarkable degree of regular scaling (Finlay et al. 2001; Yopak et al. 2010). Brain scaling simply refers to the observation that brains increased in size during evolution. Larger brains can, in principle, be due to an increase in their constituent elements or in the size of the elements: two processes that compete for the limited space available. In addition, most mammalian networks have a large intrinsic excitatory connectivity that is critical for their function (Abeles 1991; Braitenberg and Schüz 1991; Schüz and Sultan 2009) and which is, therefore, exploited to the utmost (Chklovskii et al. 2002). The upscaling of such networks also leads to an increase in the dendritic and axonal wiring required to connect larger brains, thereby causing a decrease in neuron density (Braitenberg 2001).

The mammalian brain, however, is composed of several different network architectures. One radically different type of network is found in the cerebellum. Here, the granule cell axons (the parallel fibers) show a constant length irrespective of brain size, and the granule cell density remains constant (Schüz and Sultan 2009). This difference in scaling leads to an increase in the proportion of granule cells of all neurons to 80% in humans (Andersen et al. 2003; Azevedo et al. 2009), despite the cerebellum occupying only 15% of the whole volume of the brain.

**Fig. 1:**
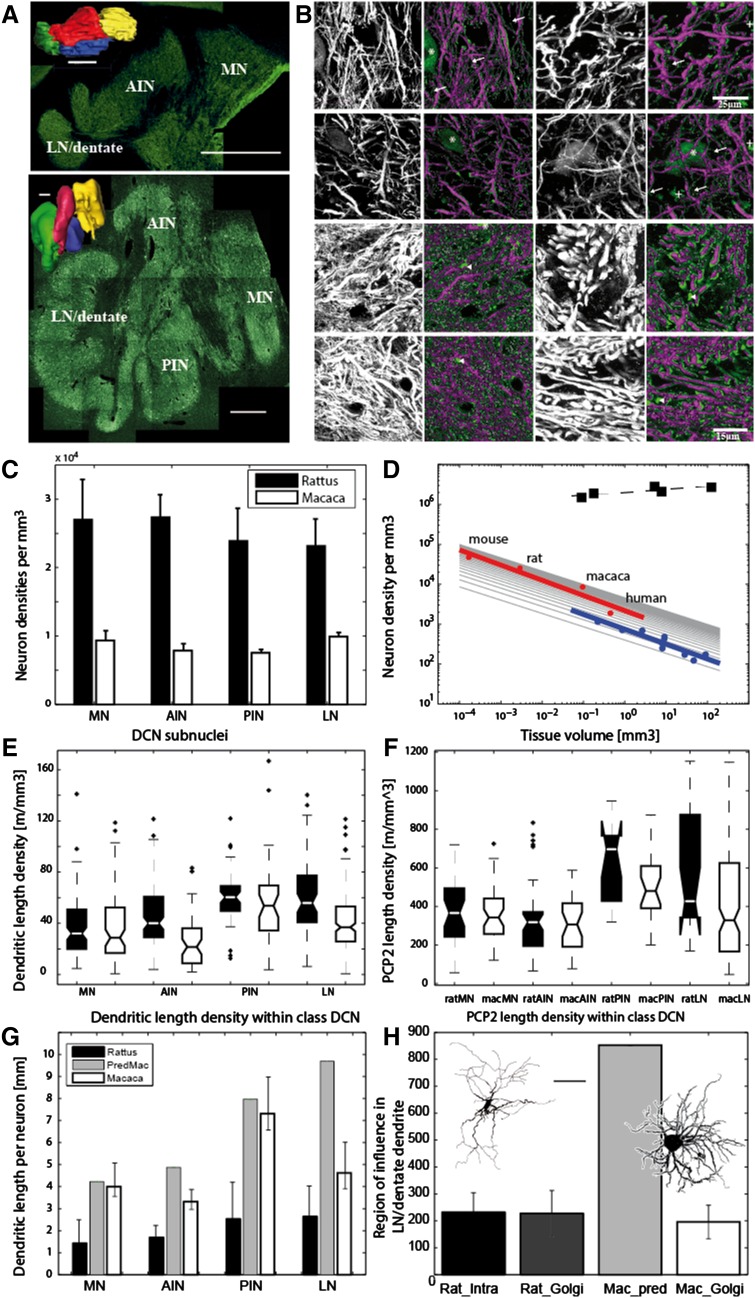
Comparison of the rat and macaque DCN. **a** Composite overviews of fluorescent MAP2 immunohistochemistry of the rat (*upper; coronal section*) and rhesus monkey (*lower; horizontal section*) DCN. Surface reconstructions of the rat (*upper*) and macaque (*lower*) DCN are also shown. The surfaces are color coded for the different DCN: MN, *yellow*; AIN, *red*; PIN, *blue* and LN/dentate, *green. Scale bars* correspond to 1 mm. **b** Examples of MAP2 and PCP2 stains acquired with the laser confocal microscope. *Black and white images* are maximal intensity projections through a stack of slices. *Magenta and green images* show the outcome of the automatic fiber and diameter reconstruction (*Magenta*) overlaid on the maximal intensity projection (*green*). The *view axis* was tilted slightly sideways to enable observation of the underlying maximal intensity projection. *Upper two rows* are MAP2 stains, while the *lower two rows* are PCP2. *Left two columns* are images from the rat and 1st and 3rd rows are from the AIN, while 2nd and 4th are from the LN/dentate. MAP2-stained somata were manually removed (marked with *asterisks*) from the reconstruction outcome. (*Plus) marks* lipofuscin that was also removed manually. *Arrow mark* subthreshold fibers and arrow heads mark other structures that the reconstruction algorithm failed to recognized. *Scale bar* for the *upper two rows* is 25 and 15 µm for the *lower two rows*. **c** Counts of neurons in rats (*n* = 4) and macaques (*n* = 2) with the optical fractionator method yielded highly significant different densities between the two species. A two-way ANOVA showed significant differences on the species level, but not on the subnuclei level (*F* value 28.3, *df* = 1, *p* < 0.0001 vs. *F* value 0.25, *df* = 3, *p* > 0.86). **d** Double logarithmic plot of the dependence of cerebellar neuron density on tissue volume. DCN (*red*) neurons show a density drop in larger brains comparable to that observed in Purkinje cells (*blue*). Regression fits showed similar results in DCN neurons and Purkinje cells (DCN: *y* = −0.38046 (±0.084) × + 3.3401; *r* ^2 ^ = 0.91, *p* < 0.05; Purkinje cells: *y* = −0.3718 (±0.053) × + 2.8728; *r* ^2 ^ = −0.86, *p* < 0.001). *Arrays of black lines* are also plotted for a comparison with a slope of −1/3. The data for cerebellar granule cells (*square, black*) which do not show a drop in density in larger brains: (*y* = 0.073435 (±0.027) × + 6.303; *r* ^2 ^ = 0.71, *p* = 0.072666) are also plotted. Sources of additional neuron densities are listed in “Material and methods”. **e, f** Quantification of fibers labeled with MAP2 (dendrites, *E*) and PCP2 (Purkinje cell axons: PCax, *F*) showed higher values for the axons. In contrast to the neuron densities, DCN classification now had an additional significant effect, thus explaining the variability of the dendrites (two-factorial ANOVA with *F* values 14.5 vs. 19.9, *df* = 1 vs. *df* = 4 and *p* < 10^−5^ vs. *p* < 10^−11^ for the factors DCN class vs. species origin) and the PCax (two-factorial ANOVA yielded *F* values 57.6 vs. 5.1 and *p* = 10^−37^ vs. *p* ≤.024 for the factors DCN class vs. species origin) in our probes. The average dendritic and PCax length densities were generally lower in the phylogenetically older DCN (i.e., the MN and the AIN), thereby contributing to the variability in the probes. **g** Comparison of the dendritic length per neuron for different subnuclei and for the two species. Dendritic length densities were normalized by neuron densities for each DCN to obtain the dendritic length per neuron. Predictions for the monkey were derived by multiplying the values obtained from the rat with the factor of volume increase to the power of 1/3 obtained from the different subnuclei. *Error bars* for the rat and monkey show the 95% confidence region obtained from the ratios of the two measured parameters (e.g., dendritic length density and neuron density). The prediction for the MN and PIN were well within the CI. The AIN prediction was somewhat higher than the CI of the monkey values. By contrast, the prediction for the LN/dentate was well above that of the CI established by our probes (only 4.8 vs. a prediction of 9.7 mm). **h** Comparison of the diameter of region-of-influence (dROI) obtained for rat and monkey dendritic trees. Rat_intra denotes dROI diameters obtained from 3D-reconstructed intracellularly filled neurons (*n* = 35) and refers to the diameters we obtain when 2D images of those neurons are analyzed by taking the area from the boundary spanned by the outer tips of the dendrites. Rat_golgi denotes diameters obtained with the same approach, but from Golgi-stained neurons (*n* = 27 for the rats; *n* = 54 for the monkey). The difference between rat and monkey (Mac_Golgi) was not significant (mean of 227 µm compared to 196 µm, respectively and *t* test *p* = 0.059). *Inlet to the left* intracellularly stained neuron from the rat. *Inlet to the right* neurons showing a small dROI with clustered dendrites from the monkey’s LN/dentate. *Scale bar* corresponds to 100 µm for the *left* and 50 µm for the *right* neuron

Precisely how this interlinkage between different scaling networks functions has not yet been established. We speculated that we could gain important insight on this question by studying the scaling between the cerebral and cerebellar cortex at their connecting hub. The DCN are a major hub which connect the cerebellar cortex with the cerebral cortex (via the thalamic nuclei). The DCN are classically subdivided into four different nuclei (Fig. 1a): the medial (MN), anterior interposed (AIN), posterior interposed (PIN), and lateral nucleus. In primates, the latter is also termed the dentate (LN/dentate). The DCN nuclei are distinguished on the basis of both their connections (Glickstein et al. 2011) and their phylogenetic development. During evolution, the DCN exhibited a remarkable change of size and form in different highly encephalized mammals. The LN/dentate increased considerably in size in primates, whereas in cetaceans the more medial PIN showed the greatest increase (Voogd and Glickstein 1998). Furthermore, the shape of the enlarged primate LN/dentate changed in a remarkable fashion to a highly folded structure, while the cetaceans’ PIN remained a globus-shaped nucleus. To comprehend the important and remarkable evolution of this major hub, we decided to quantify its major wiring components in rodents and primates.

## Materials and methods

The study supplements our previous analysis (Hamodeh et al. 2014) and now includes the data of five adult (8–14 years) male monkeys (Macaca mulatta) weighing 8–12 kg. Handling and care of the animals was approved by the regional authorities (Regierungspraesidium) and complied fully with the guidelines of the European Community (EUVD 86/609/EEC). All experiments were carried out in accordance with the institutional, national, and NIH guidelines on the use of animals in research. Following the induction of deep anesthesia (with ketamine/xylazine in rats and with barbiturates in monkeys), animals were perfused through the ascending aorta with 4% paraformaldehyde prepared in 0.1 M phosphate buffer (PB), pH = 7.4.

Histological processing was carried out as described previously (Hamodeh et al. 2014). In brief, tissue was mounted and sectioned to serial transversal slices of either 60 µm for the rats and, in the case of the monkey brains, 40 or 50 µm for immunohistochemistry and 60 µm for cell counting. For section orientation in the monkey see next chapter. The slices were then incubated in either the mouse monoclonal anti-microtubule-associated protein 2a, b (MAP2; Sigma-Aldrich, Steinheim, Germany, clone AP-20, Catalog No. 1406) or in the mouse monoclonal PCP-2 (F-3) (Cat. No. sc-137,064, Santa Cruz Biotechnology, Santa Cruz, CA). Primary antibodies were diluted at either 1:1000 (MAP2) or 1:100 (PCP2). The antibodies were visualized with the secondary antibody Alexafluor 488 goat anti-mouse (Invitrogen, Karlsruhe, Germany) at a concentration of 1:400.

### Probe acquisition and 3D reconstruction of MAP2-labeled dendrites PCP2-labeled axons

Images were acquired with a laser scanning confocal microscope (LSM 510, Carl Zeiss, Jena, Germany) using the Argon laser (wavelength 488 nm) as described elsewhere (Hamodeh et al. 2010, 2014). Image stacks were taken for each probe using a pinhole diameter equal to 1 Airy unit. Stack matrix size and other details are listed in “Supplementary material Table 1”. Probe scans were deconvoluted using AutoQuant X3 iterative “blind deconvolution” with maximum likelihood estimation and constrained iteration (Media Cybernetics, Bethesda, MD).

Probes were taken from each of the DCN in a systematic random fashion. In rats, slices were taken from every 4th section of the series. This resulted in a final spacing of 240 µm. Probes were sampled at *xy* intervals of 350 µm within a section. In monkey D98, we took every 8th horizontal cerebellar section (final slice spacing of 320 µm) and probes were sampled at an *xy* interval of 800 µm. We took sagittal sections covering the mediolateral middle parts of the LN/dentate from the other two monkeys. We obtained a total of 1041 probes: 323 (MAP2, 3 rats), 361 (MAP2, 3 monkeys), 113 (PCP2, 1 rat) and 244 (PCP2, 1 monkey).

We then used the Cavalieri estimator to calculate the volume of the DCN (VDCN) with VDCN = ∑Pi × aF × *T* × 1/ssf, with Pi being points per section, aF area per point, *T* block advance and ssf the slice sampling fraction.

Fibers were quantified by custom-written scripts (Tcl) and the Amira software package (Amira 4.1.1, Mercury Computer Systems, Chelmsford, USA). Prior to the application of our segmentation and reconstruction algorithm, preprocessing included 3D Edge-preserving-smoothing using a time-stop of 50 (in PCP2 case of 100) and step size of 5 followed by Gaussian filtering using a 5 × 5 × 5 kernel and *σ* = 1. Segmentation of the 8-bit gray-level images into a binary image was performed by testing different threshold values and then choosing a threshold of 25 for the MAP2 rat data, 40 for the MAP2 monkey data and 80 for the PCP2 case (0 black and 255 white). Threshold values were chosen such that nearby dendrites were prevented from merging. This meant that some very thin fibers became subthreshold and were, therefore, not taken into account by our algorithm. We quantified the occurrence of these subthreshold fibers in a random sub-selection of the probes. We examined 58 of the rat (18%) and 60 of the monkey dendritic probes (17%). Our selection algorithm ensured that equal numbers of probes were chosen from the different DCN. The results are summarized in Supplementary Table 2. In some instances, our reconstruction algorithm failed to recover small chunks of dendrites (Fig. 1b, arrow heads). However, this occurred rarely (at a magnitude less frequent than the subthreshold fibers) and, therefore, was not taken into account further.

The final 3D reconstruction steps included chamfer distance map calculation, thinning process and, finally, conversion to a skeleton structure as described previously (Hamodeh et al. 2010). Furthermore, we manually removed all MAP2-stained somata from the images. In the monkey probes, we also removed the lipofuscin particles. The final skeleton structure consisted of dendritic and PC axonal segments with cylinder nodes spaced at intervals of 0.5 µm along the fibers. The fiber radius is calculated at each node between the center of the node and the boundary, as obtained by the chamfer map calculation (Hamodeh et al. 2010). The reconstructed fibers were further thresholded to exclude very small particles (<0.25 µm) due to spurious labeled voxels.

### Quantitative data analysis

The following parameters were extracted from our fiber segmentation and reconstruction analysis (Hamodeh et al. 2010, 2014): dendritic and PCax diameter (*D*_dia_) and dendritic and PCax length density (Dl_dens_). The average dendritic length per neuron (Dl_neu_) was obtained by normalizing the dendritic length density by the neuron count density. We used custom-written scripts in Matlab (8.3, The MathWorks Inc., Natick, MA, 2000) to integrate the individual probes into data structures. These included the Zeiss LSM information, probes location, DCN classification, segmented fiber parameters and probe volume. We also systematically evaluated the tissue penetration of the antibody within each probe by calculating the fluorescence intensity in relation to the depth of the section. From this evaluation, and as described in detail previously (Hamodeh et al. 2010, 2014), we limited the region of fiber analysis to the stack of optical sections whose maximal intensity was 95.4% of that ascertained within the total probe. We also accounted for slice shrinkage by checking slice thickness after staining and embedding. The densities used for our analysis were obtained by multiplying each of the probes Th_ref_ with the fraction of microtome tissue block advancement and the total tissue thickness obtained after staining and embedding. Tissue thickness was measured on the Zeiss LSM 510 microscope equipped with a *z*-axis motorized stage. The regions within the DCN showed considerable shrinkage in the thickness of the section to 36% (60-µm block advancement, 22 µm after mounting), thus confirming earlier results in which we had obtained a shrinkage to 27% of the original thickness (Sultan et al. 2003).

### Neuron and glia counts

Serial Nissl slices from four rats and two monkeys (T00 and B99) were used for counting neurons in the DCN. Purkinje cells in the cerebellar cortex were also counted for T00. Cells were counted using the optical fractionator (MicroBrightField Inc., Williston, VT, USA) as described previously (Hamodeh et al. 2014). On account of their small and homogenously dark nuclei lacking surrounding cytoplasm, the glia cells could be readily distinguished from neurons, the latter having lightly stained nuclei with a nucleolus and surrounding cytoplasm with Nissl bodies. In the case of the rat DCN, we used a counting frame of 50 × 50 µm with spacing of 200 × 200 µm and counted every 4th slice. In the monkey DCN, we used a spacing of 400 × 400 µm and took every 8th slice into account. Dissector height was set at 14 µm for the rats and 25 µm for the monkey with a 2-µm guard zones. This counting procedure yielded a coefficient of error (CE) of below 0.05 for both species. Section thickness was measured in each probe and found to be on average 23.4 µm (std = 2.7 µm) in the rats and 29.6 µm (std = 0.7 µm) in the monkeys. Neuron numbers were obtained by calculating *N* = Σ*Q* × 1/ssf × 1/asf × 1/hsf, where Σ*Q* is the total counted neuron number, ssf the slice sampling fraction, asf the ratio between frame area and the area of the sampling grid, and hsf the height sampling fraction (ratio of dissector height and section thickness). Additional DCN and Purkinje cell counts for other mammals were obtained from earlier studies (Andersen et al. 1992; Korbo et al. 1993; Mwamengele et al. 1993; Sultan et al. 2002; Schüz and Sultan 2009).

### Estimating dendritic region-of-influence

The dendritic region of influence (dROI) was estimated for the rat and the monkey LN/dentate from two sources. For the rat, we compared the dROI from a previous 3D dendritic reconstruction (Sultan et al. 2003) based on neurons filled intracellularly with neurobiotin with calibrated drawings of Golgi-stained neurons (Chan-Palay 1977). The dROIs in the 3D reconstructions were calculated with Matlab’s built-in function convhull. We compared these 3D estimates with 2D estimates of the same neurons, but now based on measuring the area of the polygon that connects the outer tips of the dendritic tree from 2D plots (from Fig. 5 in Sultan et al. 2003). This comparison of the 3D and 2D data yielded similar results (232 ± 73 vs. 224 ± 75 µm, respectively) and a *t* test showed no statistical difference (*t* = −0.48; *df* = 24; *p* = 0.63). We, therefore, obtained further estimates from calibrated drawings of Golgi-stained neurons (Chan-Palay 1977) for the rat and the monkey by measuring the area of the polygon that connects the outer tips of the dendritic tree. Supplementary material Fig. 1 shows examples of such polygon from the two sources. Finally, we obtained the dROI diameters from the polygon measurement by taking a circle diameter occupying the area (dROI diam = 2*(polygon area/Pi)^0.5).

### Statistical analysis

Statistical analysis was performed within Matlab with custom-made scripts and built-in routines. Univariate data were plotted using the boxplot function, with five horizontal lines indicating the 10th, 25th, 50th, 75th, and 90th percentiles. The notches display the 95% confidence intervals for the median. Statistical significance within the group was tested with two-way ANOVAs (for difference within species and DCN classification). Estimates of the dendritic length per neuron were obtained as ratios of two measured parameters and were checked for significant differences by comparing their confidence intervals (Donner and Zou 2012). In a similar fashion, confidence intervals (CI) comparing the difference between fiber distributions were calculated as confidence level = *µ*_i1−_ *µ*_i2_ ± *t*_1 − α/2,ni1+ni2−2_ * (2*MSE_i_/*n*_i12_)^0.5^, with *µ*_i1_ and *µ*_i2_, the means at the *i*_th_ *b*_in_, *n*_i1_, *n*_i2_ the sample size at the *i*_th_ *b*_in_, *n*_i12_ the joint sample size calculated as 2/(1/*n*_i1_ + 1/*n*_i2_) and MSE_i_ the estimated standard error calculated as (sd_i1_^2^ + sd_i2_^2^)/2. Other statistical differences were tested with the Student *t* tests. Linear fits were performed using the Matlab robustfit routine.

## Results

### Regular scaling of neuron density and wiring in the DCN

We analyzed the Purkinje cell axons (PCax) and the dendrites of the DCN neurons, comparing the rat and rhesus monkey DCN in a systematic and quantitative fashion. We used fiber reconstruction and tracking algorithms (Fouard et al. 2006; Hamodeh et al. 2010, 2014) on immunofluorescent stained material (Fig. 1a, b), which then enabled us to semi-automatically extract wiring information on fiber length density and fiber diameters. We began by comparing the fiber density estimates with neuron density estimates in the different DCN. Our results show that the classification of the DCN does little to explain the neuron density variability in our samples. By contrast, and as anticipated, the species classification has a major effect on explaining the variability (two-factorial ANOVA with *F* values 0.25 vs. 23.4 with *p* < 0.0001 vs. *p* = 0.86 for the factors DCN class vs. species origin). The lower neuron density in the rhesus monkey corresponds to a regular down-scaling of neuron number in larger brained animals (Fig. 1c, d) and is comparable to the scaling observed in Purkinje cell numbers. Our data for the DCN, therefore, lie within the range of the theoretically expected scaling of neuron number depending on tissue volume raised to the power of −1/3 (Braitenberg 2001).

We detected a larger influence of the DCN classification on the variability of the dendritic and axonal wiring than on the variability of neuron densities (Fig. 1e). A two-factorial ANOVA showed a major influence of DCN classification on the dendritic length density variability (two-factorial ANOVA with *F* values 14.5 vs. 19.9, *df* = 1 vs. *df* = 4 and *p* < 10^−5^ vs. *p* < 10^−11 ^for the factors DCN class vs. species origin). A similar major influence of DCN classification was also observed in the variability of the PCax (Fig. 1f), where a two-factorial ANOVA yielded *f* values 57.6 vs. 5.1 and *p* = 10^−37 ^ vs. *p* ≤.024 for the factors DCN class vs. species origin. One major influence in accounting for the variability in the DCN wiring is the higher number of dendrites and PCax length density in the PIN and the LN, the phylogenetically newer DCN (Hamodeh et al. 2014). In general, the DCN have a lower fiber length density than for instance the cerebral cortex. This has been described previously (see Hamodeh et al. 2014).

### Normalizing dendritic density by cell number: hypometric dentate dendritic trees

In a next step, we examined the dendritic length density normalized by the neuron densities, yielding the average amount of dendritic length per neuron (Fig. 1g). As already reported previously (Hamodeh et al. 2014), by comparing our population-based approach with results obtained from intracellularly stained neurons of the rat LN/dentate (Sultan et al. 2003), we obtained good agreement for the amount of dendrite per neuron (2.83 compared with 2.65 mm). As anticipated, we obtained a larger amount of dendrites per neuron (rat: 2.1 vs. monkey: 4.8 mm) in the rhesus monkey DCN. We compared the two species by scaling the rodent data by a factor derived from the individual DCN volumes to the power of 1/3. In the case of MN and PIN, the predictions are well within the confidence intervals obtained for the primate estimate. However, in the case of the primate LN/dentate, the prediction for a nucleus of its size should be in the region of 10 mm of dendritic length per neuron. We obtained a length of only 4.8 mm; about 50% less than anticipated.

To validate our findings, we compared them to a different estimate of dendritic tree size by examining the region-of-influence (dROI) defined as the polygon drawn by connecting the distant dendritic distal tips. We compared the data obtained in a previous study by intracellularly filling LN/dentate neurons in rats (Sultan et al. 2003) with Golgi-stained neurons of rats and rhesus monkeys (Chan-Palay 1977). The comparison of the differently stained rat LN/dentate neurons yielded a similar dROI diameter. The prediction for the monkey based on the LN/dentate tissue volume scaling yielded a diameter of ~850 µm. Our measurement, however, was much smaller than expected (190 µm). This is in agreement with the dendritic length per neuron result of our population-based analysis, which was also smaller than expected, and indicates that the majority of neurons in the primate LN/dentate must exhibit a hyposcaling with dwarf-like clustered dendrites (Fig. 1h, figure inlets).

We went on to estimate the potential bias in our reconstruction algorithm caused by the very thin fibers being subthreshold (see Supplementary Table 2). An ANOVA showed no significant effect on the amount excluded due to DCN or species classification. Therefore, our finding of hyposcaling dendrites in the primate LN/dentate is not explained by this effect.

We also compared the PCax length density to the number of Purkinje cells in the rat and rhesus monkey. By normalizing the total PCax length of the DCN (see Supplementary data: estimating PCax length in rats and rhesus monkey), we obtained 7.1-mm PCax length per Purkinje cell for the rats as opposed to 25.1 mm for the rhesus monkey. This is comparable to the rhesus monkey prediction of 27.9 mm based on the factor derived from DCN volumes to the power of 1/3.

### Scaling of dendritic and axonal diameters

**Fig. 2:**
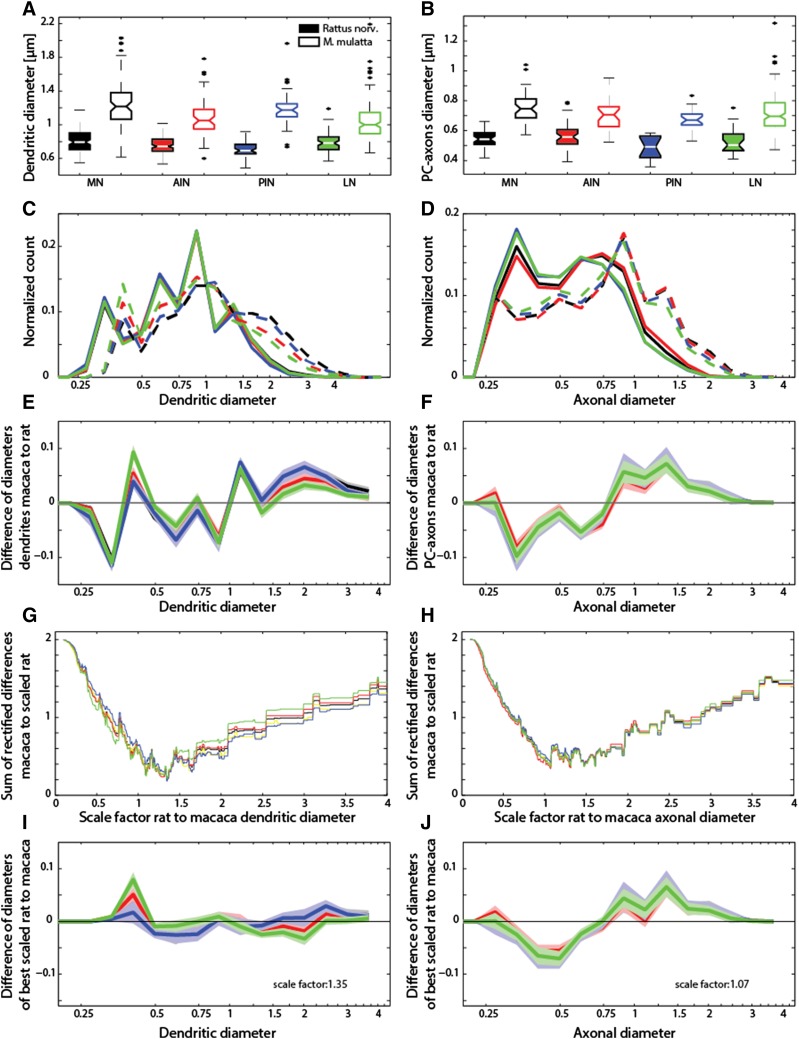
Fiber diameter in different DCN subnuclei compared to predictions. **a** Monkey DCN dendrites exhibited larger diameters than rats. The thickest dendrites were measured within the monkey MN. The largest difference was observed within the PIN (factor of 1.7 between monkey and rats), with 1.19 µm (sd = 0.22) in monkeys compared to 0.7 µm in rats (sd = 0.08). The second largest difference was observed in the MN, (×1.5), with 1.25 µm (sd = 0.3) in monkeys compared to 0.81 µm in rats (sd = 0.14). The LN/dentate showed the smallest difference (1.3), with 1 µm (sd = 0.23) in monkeys compared to 0.79 µm in rats (sd = 0.12). Statistical analysis with a two way-factorial ANOVA showed that both the influence of the species (*F* = 511; *p* < 0.0001), and the origin of the probes from the different subnuclei (*F* = 8.76; *p* < 0.0001) were highly significant. **b** As in the dendritic diameters, differences were also observed in the PC axons between the two species. Again, the MN showed the largest increase from 0.54 to 0.75 µm. The differences were statistically significant (two-way ANOVA yielded *F* = 201.9, *p* < 10^–36^ for species vs. *F* = 8.5; *p* < 10^–6^ nucleus). **c, d** Histograms showing diameter distribution for the dendrites (**c**) and PCax (**d**) for different nuclei (*color coded*) and for the rats (*continuous line*) and monkey (*dashed line*). The dendritic diameters (C) of the monkey are shifted to larger diameters. Within the rats, the different DCN are indistinguishable from each other. By contrast, larger differences between the DCN are found in the monkey: the LN/dentate has more of the smaller diameter dendrites (~0.4 μm) and fewer thick dendrites (>1.5 μm). A similar, but smaller pattern is observed in the AIN. Histograms were normalized by the sum of all diameter counts for the respective species and subnucleus. Fiber diameters are plotted as natural logarithms. **e** Difference calculation between the rhesus monkey and rat dendritic histograms (**c**) for each DCN. The difference calculation is plotted together with the 99% CI (*lighter shaded color*). The curves overlie each other and are within their CI up to a diameter of 0.4 µm, at which point the monkey dendritic histogram for the LN/dentate exceeds the others. **f** Difference calculation between the rhesus monkey and rat PCax histograms (**d**) for each DCN. As in **e**, the difference calculation is plotted together with the 99% CI. **g, h** Sum of the rectified difference curves obtained for different scaling factors. The rat fiber diameters were multiplied with varying scaling factors (scaling factors plotted on the *abscissa*) and then subtracted from the primate data. The difference was rectified and summed and plotted on the ordinate axis (**g** dendrites and **h** PCax). **i, j** Results for best scaling factors (**i** dendrites and **j** PCax). In the case of the dendrites, a scale factor of 1.35 yielded optimal scaling. A wide range of values (1–1.5) yielded similar results in the case of the PCax, i.e., with little difference between the DCN. The optimal dendritic scaling factor, however, also showed an excess of thin dendrites mainly for the monkey LN/dentate and the number of dendritic diameters around 2 µm was lower than predicted. *Color code* for DCN: MN: *black*; PIN: *blue*; AIN: *red*; NL/dentate: *green*

Our fiber tracking and reconstruction algorithm also estimated the diameter of the stained dendrites and PCax. As predicted, we found larger diameter estimates for both fibers in the monkey (Fig. 2a, b). An ANOVA analysis shows that the dendritic diameter variability was largely due to differences in species (*F* = 498; *p* < 0.0001) and, to a smaller degree, to the nuclear origin (*F* = 17.5; *p* < 0.0001). In the PCax diameters, we also found a larger influence of species (*F* = 201.9, *p* < 0.0001) versus nucleus (*F* = 8.5; *p* < 0.0001).

A comparison of the fiber diameter histograms (Fig. 2c, d) shows that the increase in size differs between the dendrites and axons: within the dendrite, there appears to be a general shift to higher diameters in primates. By contrast, the diameter range in axons broadens to include larger diameter axons in primates. For further analyses, we first examined how the diameters were distributed in the two species by subtracting them bin by bin for each DCN (Fig. 2e, f). We confirm that the primate dendritic diameters in the LN/dentate differ from the other distributions around the diameters 0.4 µm, based on the fact that they do not overlap with the 99% confidence intervals (CI) of the other distributions. We then compared a primate and an upscaled rat diameter distribution. Fig. 2g, h shows the sum of the rectified differences between the distributions (upscaled rat vs. primate) for different scaling factors. Although the two figures show certain similarities (steep descent to factor 1 and gradual rise for values larger than 1.5), the difference becomes evident in Fig. 2i, j. Here, we plotted the optimal scaling factor (yielding the smallest difference between primate and upscaled rat). With regard to the dendrites, a scaling factor of 1.35 yielded a near-perfect match between the upscaled rat and the primate. As for the axons, a broad range of scaling (1–1.5) yielded similar results, with a non-optimal fit between primates and upscaled rat pointing to a more complex scaling in axons. Such complexity was to be expected since the PCax contains both the myelinated and the unmyelinated axon portions within the DCN. A similar distribution pattern can be observed for cortical axons (Wang et al. 2008; Buzsaki and Mizuseki 2014).

On the basis of our observation of hypometric scaling of the dendritic length in the primate LN/dentate, we would have expected a specific change in the dendritic diameter distribution in this nucleus. In a detailed examination of the different DCN histograms, we observed that, unlike in the axons, there was a deviation in the dendritic LN/dentate distribution (Fig. 2e), with a high proportion of small diameters (around 0.4 µm), and a lower proportion of large diameters (around 2 µm) in the monkey samples. Such a difference would arise if an isodendritic branching pattern changes to an idiodendritic pattern (Sultan et al. 2001), the former having more primary dendrites and the latter more distal bifurcations (yielding more thin dendrites). An earlier quantitative analysis conducted on the rodent LN/dentate neurons revealed a heterogeneous population due to the presence of two such extreme branching patterns (Sultan et al. 2001).

In contrast to our findings in the dendrites, the different DCN histograms of the PCax did not show such differences between the different DCN for the respective species. This also supports our interpretation that differences in the monkey dendritic distributions are not due to biases introduced by our reconstruction algorithm.

**Fig. 3:**
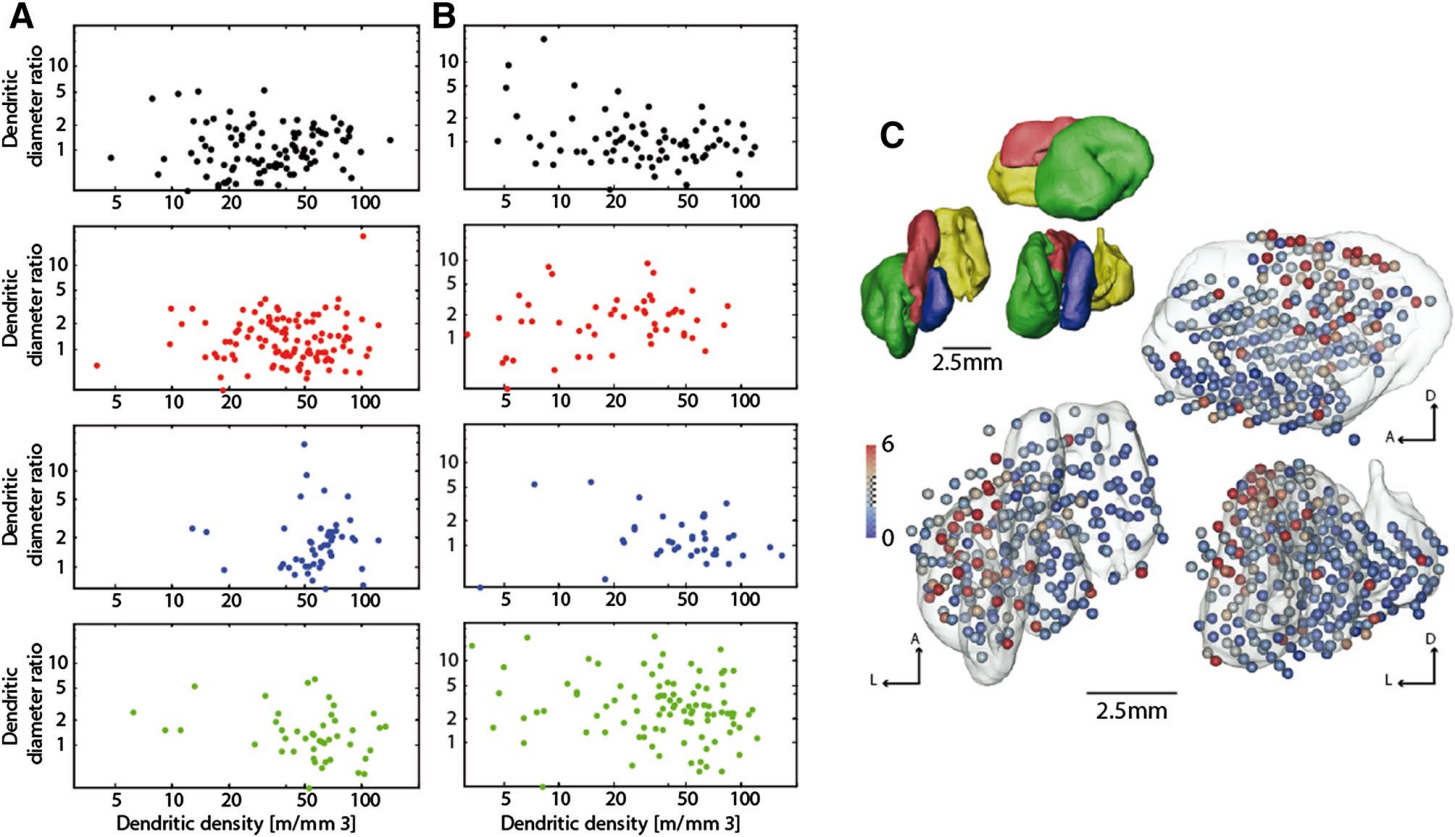
Dendritic diameter ratio. **a, b** Double logarithmic plot of dendritic diameter ratio vs. dendritic length density for the rat (**a**) and the rhesus monkey (**b**). The four vertical subplots are the data for the MN (*black*), AIN (*red*), PIN (*blue*) and the LN/dentate (*green*). The diameter ratio was taken between thin diameters (rat: 0.35; monkey: 0.41 µm) and the thick diameters (rat: 1.35; monkey: 2 µm). The PIN and LN generally showed higher dendritic length densities on average. In the primate LN/dentate, a larger number of probes showed a higher dendritic diameter ratios than in the rat (**b**, *lower panel*). An ANOVA test showed significant effect of species and DCN classification for the dendritic diameter ratio (*F*-stat 6.79, *DF* = 1, *p* < 0.01 and *F*-stat: 8.68, *DF* = 4, *p* < 0.0001 for species and DCN classification, respectively). A post hoc test ascertained that the primate LN/dentate had a significantly higher diameter ratio than the other subnuclei (post hoc hsd test comparing monkey LN to AIN: *p* < 0.01; LN to NM: *p* < 0.0001; LN/ dentate to PIN: *p* < 0.0005). **c** Surface models of the rhesus monkey DCN and the dendritic diameter ratio. The *upper left* models are surface renditions with the different subnuclei color coded: MN (*yellow*), AIN (*red*), PIN (*blue*) and the LN/dentate (*green*). The *left* shows the view from dorsal, the *upper right* from lateral, and the *lower right* from posterior. The same views are shown again in the larger 3D model version with transparent DCN surfaces. In addition, we plotted the distal–proximal dendritic diameter ratio as the proportion of 0.41-µm diameters divided by the proportion of 2-µm diameters color coded on small spheres, with *red* for larger ratios (more small diameters) and *blue colors* for lower ratios (larger thicker diameters)

We also derived a parameter to better capture the differences in the dendritic diameter histograms. We took the ratio of the small dendritic diameter counts (at 0.41 µm) of the rhesus monkey and divided these by the counts at larger diameters (at 2 µm). In the rats, these corresponded to the diameters 0.35 and 1.35 µm, respectively (Fig. 2e). In Fig. 3a, b, we plotted this ratio against the dendritic density to test whether higher dendritic densities lead to thinner dendrites and higher ratios. We found no significant correlation between the two parameters. Furthermore, we found a similar pattern for most of the DCN in the two species with the exception of the LN/dentate. We used a two-way ANOVA to test the variance explained by species and DCN classification and discovered that the two factors (*F*-stat 6.79, *DF* = 1, *p* < 0.01 and *F*-stat: 8.68, *DF* = 4, *p* < 0.0001 for species and DCN classification, respectively) had a statistically significant influence. We also performed a post hoc test and ascertained that the primate LN/dentate had a significantly higher diameter ratio than all the other subnuclei (post hoc HSD test: LN to AIN: *p* < 0.01; LN to NM: *p* < 0.0001; LN/dentate to PIN: *p* < 0.0005). Furthermore, we plotted the diameter ratio on a 3D-surface rendering of the primate DCN (Fig. 3c) and found that the higher dendritic diameter ratios within the LN/dentate tended to be located in the dorsal region.

## Discussion

In summary, we observed a specific deviation from regular scaling in the dendrites of the LN/dentate of the primate brain. We observed a deviation from scaling in the dendritic length per neuron, together with a smaller than predicted dendritic region-of-influence in the LN/dentate neurons. These deviations in scaling support the notion that the primate brain has a special architecture and represents a distinct cerebrotype (Haug 1970; Clark et al. 2001; Watson et al. 2012). The presence of neurons with restricted and clustered branching trees explains our findings of special dendritic scaling. These neurons were first described qualitatively by Chan-Palay (1977) with Golgi staining and later quantitatively with intracellular staining techniques (Sultan et al. 2003). The increased presence of such neurons is also supported by our observation of specific changes in the dendritic diameter distribution. Such dendritic adaptations could, in principle, be either due to adaptations in the connectivity of the network or to a change in the computational role of the neurons. It has already been proposed that clustered/idiodendritic branching (Ramon-Moliner and Nauta 1966) allows for more interaction between the synaptic inputs simply because the synapses are closer together (Koch et al. 1983). This closer interaction could, then, be more effective in eliciting the well-known rebound bursts of DCN neurons following Purkinje cell inhibitory inputs (Llinas and Muhlethaler 1988; Aizenman and Linden 1999). However, such a rebound burst is also present in the other DCN neurons (Aizenman and Linden 1999) and is, therefore, in no way unique to the LN/dentate. This agrees with the general view that the computational operations within the cerebellum are the same in the different cerebellar subregions (De Zeeuw et al. 2011). The change in network connectivity, therefore, continues to be the principal explanation of the observed dendritic changes.

A further consequence of the smaller dROIs of the primate LN/dentate would be that more independent modules could be packed within the LN/dentate. Our results predict that the smaller dROIs (by a linear factor of about ~4.5 smaller than expected) allow for 90x more modules to be placed within the LN/dentate volume. We could expect an even larger increase for the ape and human dentate, with an even more flattened LN/dentate sheet (Sultan et al. 2010). The advantage of such an increase in the number of computational modules is, however, not yet clear. Different views on the role of the expanded primate cerebellum and LN/dentate range from sensorimotor elaboration of finger use or eye–hand coordination (Bower 1997; Glickstein et al. 2005) to non-motor cognitive contributions (Middleton and Strick 1997; Stoodley and Schmahmann 2009). On the basis of these two views, it could be predicted that more cerebellar modules are required for the finer scaled and more versatile finger movement, or for more combinations of hand and eye modules. Alternatively, more non-motor cognitive modules are required for the vast multitude of human behavior. Either way, our findings provide an additional handle to tackle the long-debated role of the human LN/dentate and could enable us to directly relate crucial evolutionary adaptations to the unique morphology of the primate LN/dentate.

Another important implication of our observations concerns the well-established parasagittal organization of the cerebellum (Glickstein et al. 2011), which is probably related to different internal models (Wolpert et al. 1998). An increase in the number of modules might imply an increase in the number of parasagittal strips in the hemisphere connected to the LN/dentate, which would confirm a recent prediction (Jörntell 2017). In principle, one could expect the increase in the number of modules to occur along one of the two main cerebellar axes: the medio-lateral or the antero-posterior cerebellar axis. However, the larger surface increase of the dentate in the mediolateral axis (Sultan et al. 2010) would provide more space to accommodate independent modules, which would, in turn, support the theory of increased parasagittal stripes.

Our approach is based on the analysis of the overall neuronal population of the DCN and, as such, does not allow us to draw conclusions about the different neuron class composition within the DCN. However, we were able to utilize this approach to extend our analysis beyond the rodent species to that of primates, in which it is challenging to acquire sufficient information about individual neurons. In addition, the unbiased approach used in our study can be difficult to achieve with Golgi staining or intracellular staining (Hamodeh et al. 2014). Our population approach nevertheless enabled us to detect changes in the dendritic bifurcation pattern of DCN neurons.

In summary, our analysis of the major hubs connecting the cerebellar with the cerebral cortex verifies that these hubs can show changes from a predictable scaling (neuron density decrease and dendritic/axonal length increase) to a different scaling mode, with smaller dendritic ROIs allowing for a larger number of modules in the most enlarged DCN of primates, in the LN/dentate. This surprising result is well in keeping with the long-known observation of the unique anatomy of the primate LN/dentate from which its name is derived: the flattening and folding of its gray matter into a tooth-shaped structure (Voogd 2003; Tellmann et al. 2015). It has already been suggested that the LN/dentate dendritic adaptations lead to reduced mechanical tension with the DCN, thus allowing it to flatten (Sultan et al. 2003), as was already proposed for the cerebral cortex (Van Essen 1997). Mapping the dendritic diameter ratio associated with this dendritic adaptation to the dorsal LN/dentate also agrees well with this proposal, since this so-called microgyric (dorsal) region (Voogd 2003) (Tellmann et al. 2015) shows marked flattening in the macaque (Sultan et al. 2010). Our result of the preponderance of clustered dendrites trees in the primate LN/ dentate not only confirms Chan-Palay’s earlier description of these neurons (Chan-Palay 1977) but is also the first to relate their special branching pattern to the unique morphology of the primate LN/dentate.

## Electronic supplementary material

Below is the link to the electronic supplementary material.

Supplementary material 1 (DOCX 22420 KB)

## Abbreviations

AIN: Anterior interposed nucleus
asf: Area sampling fraction
CI: Confidence interval
*D*_dia_: Dendritic diameter
Dl_dens_: Dendritic length density
Dl_neu_: Dendritic length per neuron
dROI: Dendritic regions of influence
hsf: Height sampling fraction
LN/dentate: Lateral nucleus/dentate nucleus
LVN: Lateral vestibular nucleus
MAP2: Microtubule-associated protein 2a, b
MN: Medial nucleus
PIN: Posterior part of interposed nucleus
PCax: PCP2-stained Purkinje cell axons
PCP2: Purkinje cell-specific protein 2
Σ*Q*: Total counted neuron/glia number
ssf: Slice sampling fraction
Th_ref_: Section depth used to calculate fiber densities within 3D-QIHC probes
*V*_DCN_: Volume of DCN per side
Vf_dend_: Volume fraction of dendrites
