# Supplementary material for: Uncovering specific changes in network wiring underlying the primate cerebrotype
Source: Brain Struct Funct. 2017 Mar 25;222(7):3255–66. doi: 10.1007/s00429-017-1402-6 (PMC5585288; doi:10.1007/s00429-017-1402-6)
Supplement: Supplementary file 1 — Supplementary material 1 (DOCX 22420 KB) [file 429_2017_1402_MOESM1_ESM.docx]

# Supplementary material

### TITLE: Uncovering specific changes in network wiring underlying the primate cerebrotype.

### AUTHORS: Salah Hamodeh, Ayse Bozkurt, Haian Mao and Fahad Sultan

## Supplementary methods

### Supplementary Table 1

|  | Matrix size (xy) | Z-stack size | Pixel size (xy) in µm | Z- optical slice thickness (µm) |
| --- | --- | --- | --- | --- |
| Rat (MAP2) | 512x512 | 41 (±11) | 0.11-0.15 | 0.31 |
| Rat (PCP2) | 512x512 | 18 (±3) | 0.093 | 0.35 |
| Monkey (MAP2) | 512x512 | 67 (±20) | 0.146 | 0.41 |
| Monkey (PCP2) | 512x512 | 35 (±5) | 0.093 | 0.32 |

### Supplementary Table 2: missing thin dendrites

| **DCN classification** | **Rat (mean ± sd)** | **Monkey** | **ANOVA between DCN (F-value; df; p)** | **ANOVA between species (F-value; df; p)** |
| --- | --- | --- | --- | --- |
|  | 0.114% | 0.1265% | 0.89; 3; p>0.44 | 0,07; 1; p> 0.79 |
| **FN** | 0.16 ±0.14 | 0.13 ±0.17 |  |  |
| **AIN** | 0.11 ±0.06 | 0.22 ±0.63 |  |  |
| **PIN** | 0.075 ±0.04 | 0.08 ±0.15 |  |  |
| **LN/dentate** | 0.11 ±0.06 | 0.07 ±0.06 |  |  |

### Supplementary figure 1: Estimating dendritic region-of-influence


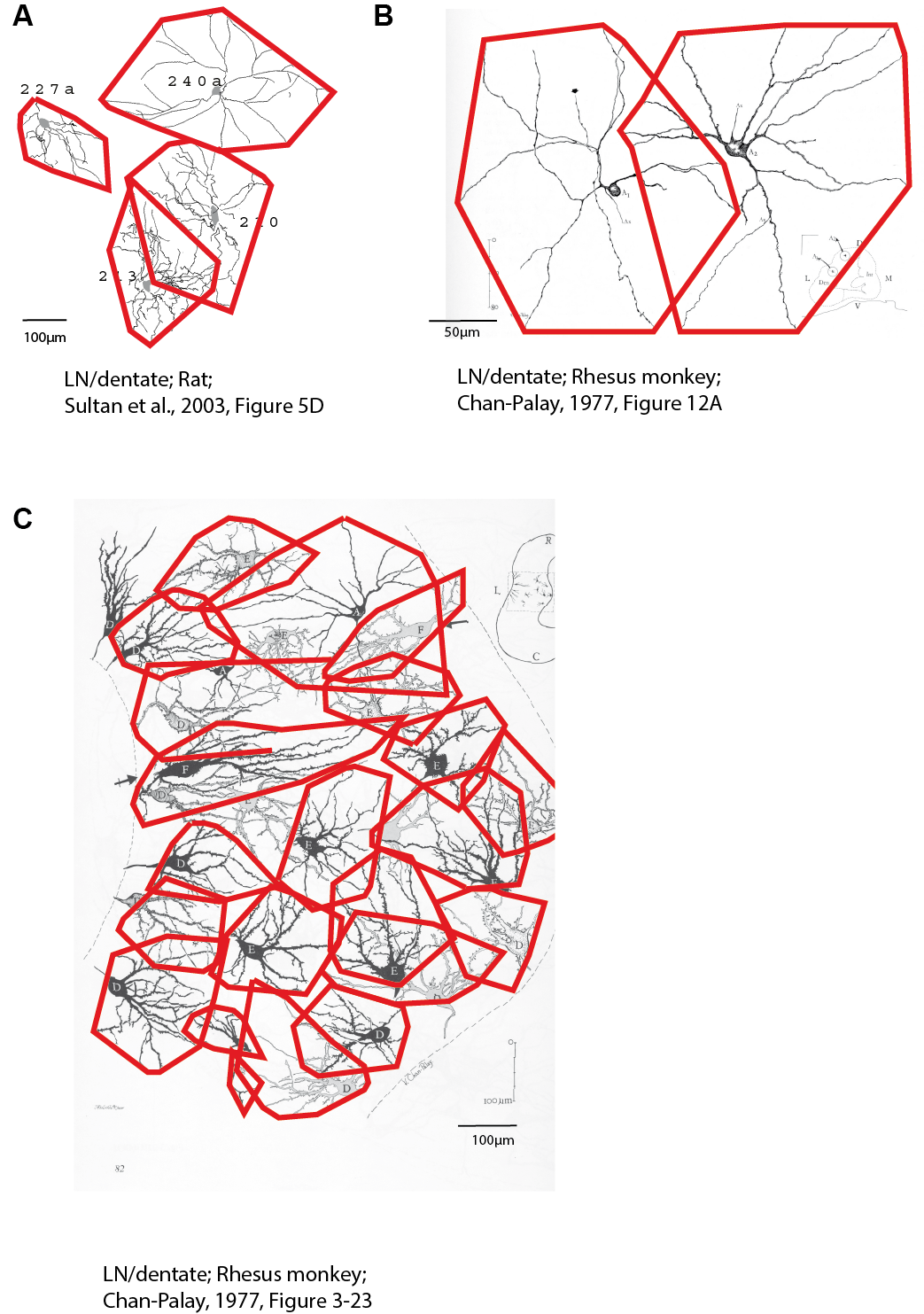


Two sources were used to estimate the dendritic region of influence (dROI) for the rat and monkey LN/dentate. For the rat LN/dentate, we used 3D dendritic reconstruction ([Sultan et al., 2003](#_ENREF_33)) based on neurons filled intracellularly with neurobiotin (examples shown in **A**). We compared the data from those results with calibrated drawings of rat (**B**) and monkey (**C**) Golgi-stained neurons ([Chan-Palay, 1977](#_ENREF_11)). The polygons depicted connect the outer tips of the dendrites (red polygons).

### Supplementary figure 2:


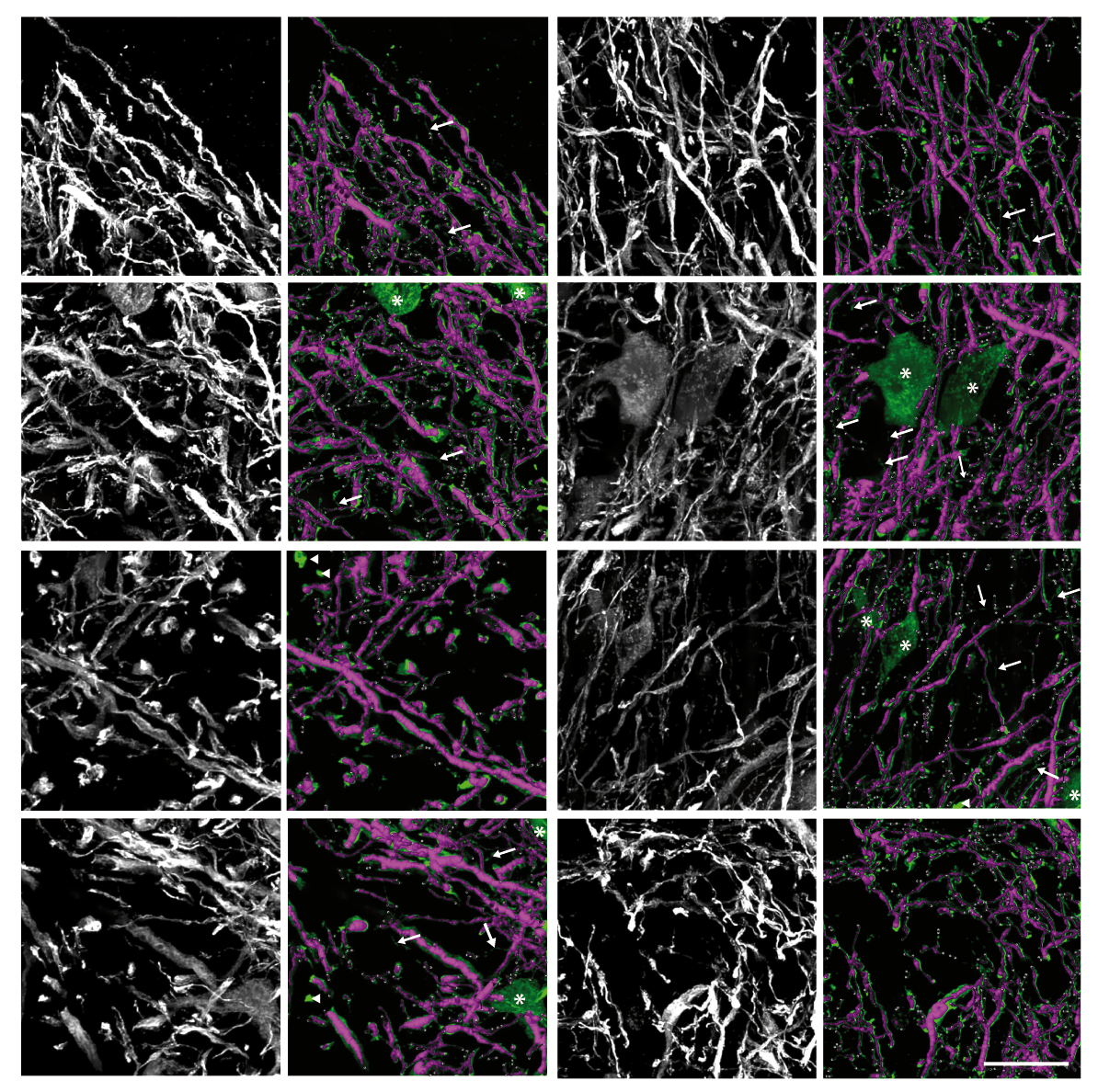


Further examples of fiber reconstructions from rat (MAP2). First two columns on the left are from MN. Third and fourth column are from the AIN. First and third column are maximal intensity projections through laser confocal stack images, while second and fourth are fiber reconstruction results (magenta) overlaid on maximal intensity projections (green). Asterisks mark manually removed MAP2-stained cell bodies. Arrows mark thin dendrites that were subthresholded to our reconstruction algorithm. Arrow head mark fibers that failed to be detected by the algorithm. Scale bar: 25µm.

### Supplementary figure 3:


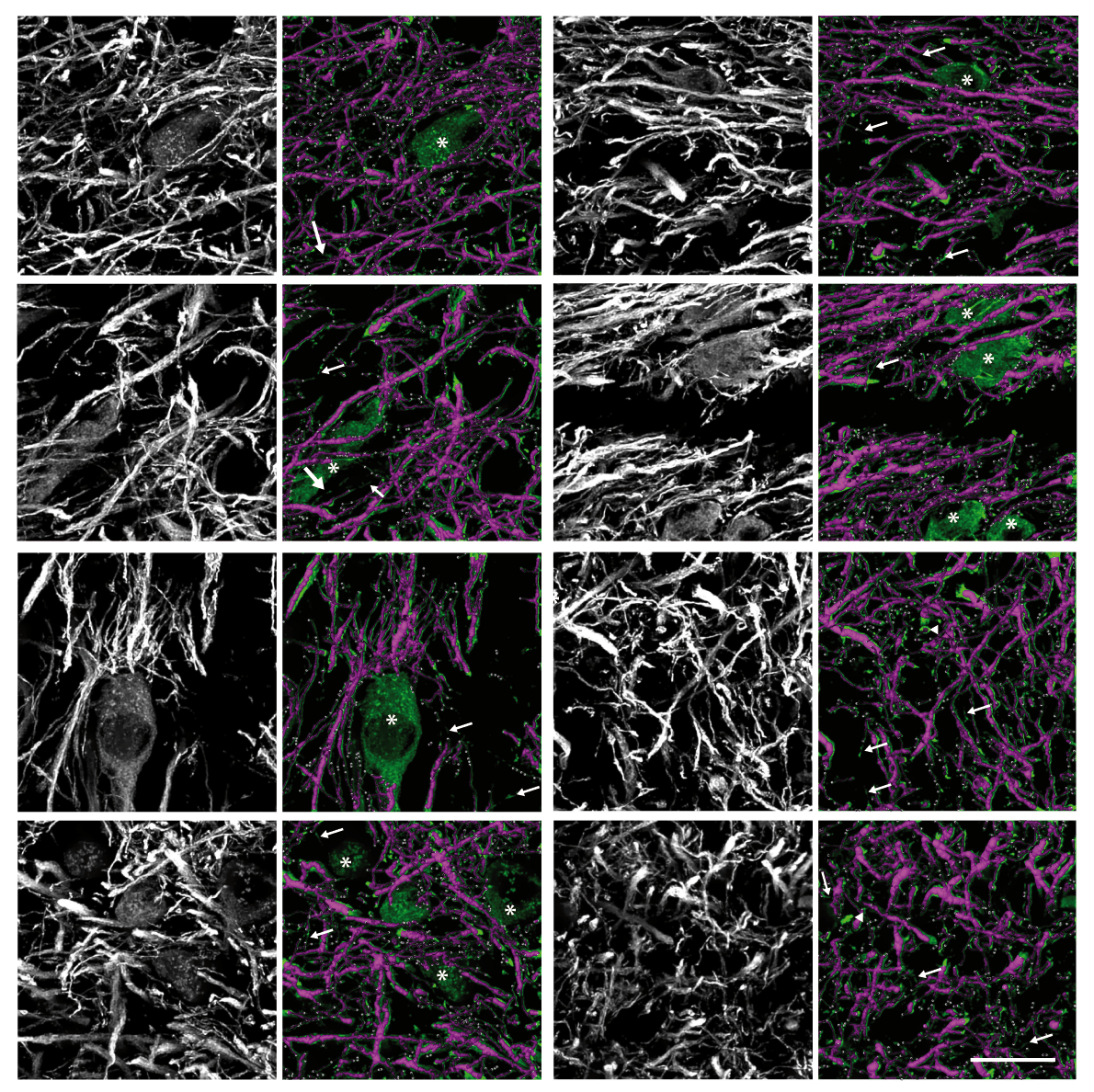


Additional examples of fiber reconstructions from the rat (MAP2). First two columns on the left are from PIN. Third and fourth column are from the LN/dentate. Scale bar: 25µm.

### Supplementary figure 4:


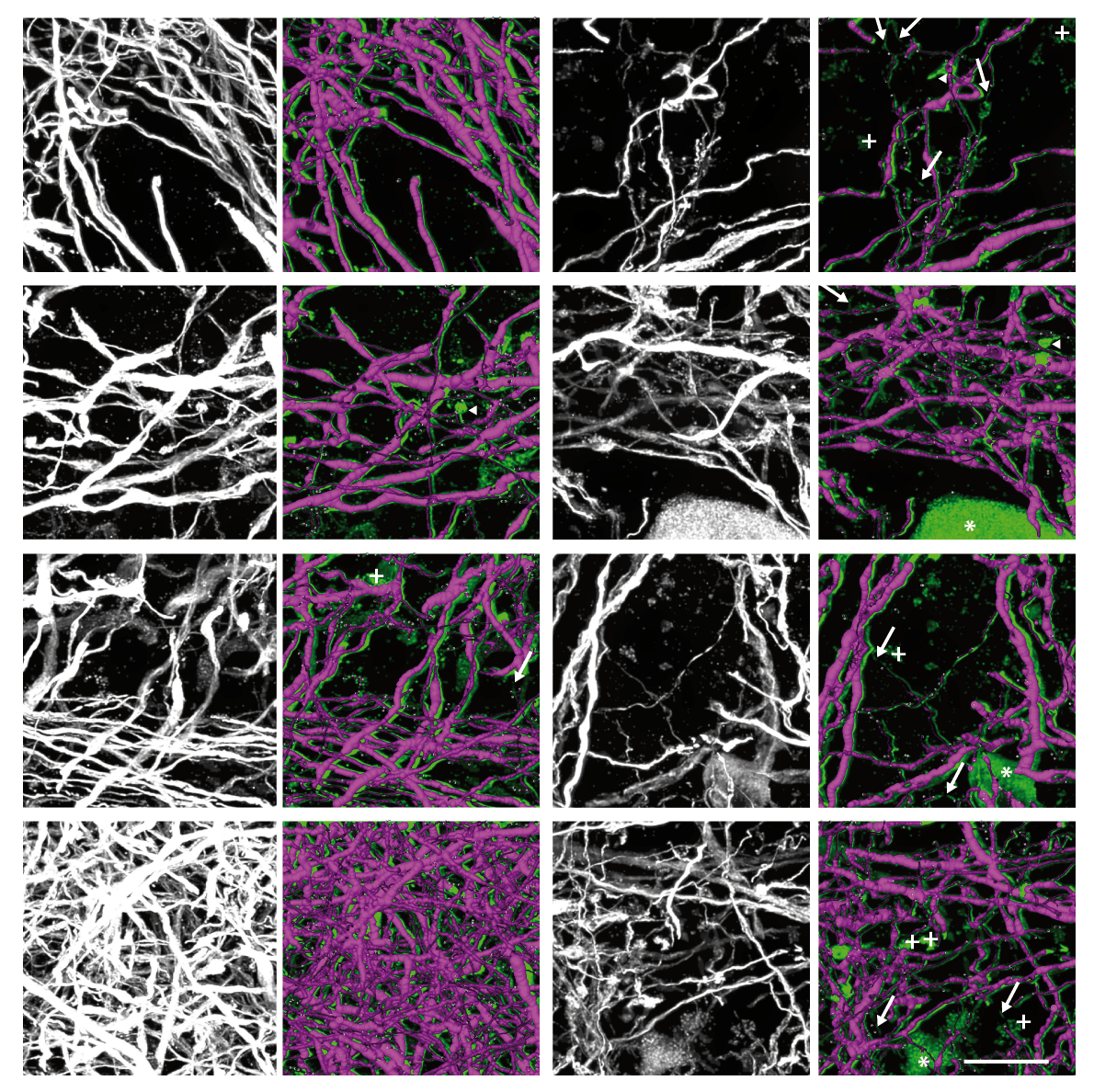


Additional examples of fiber reconstructions from the monkey (MAP2). First two columns on the left are from MN. Third and fourth column are from the AIN. ‘+’ marks manually removed lipofuscin particles. Scale bar: 25µm.

### Supplementary figure 5:


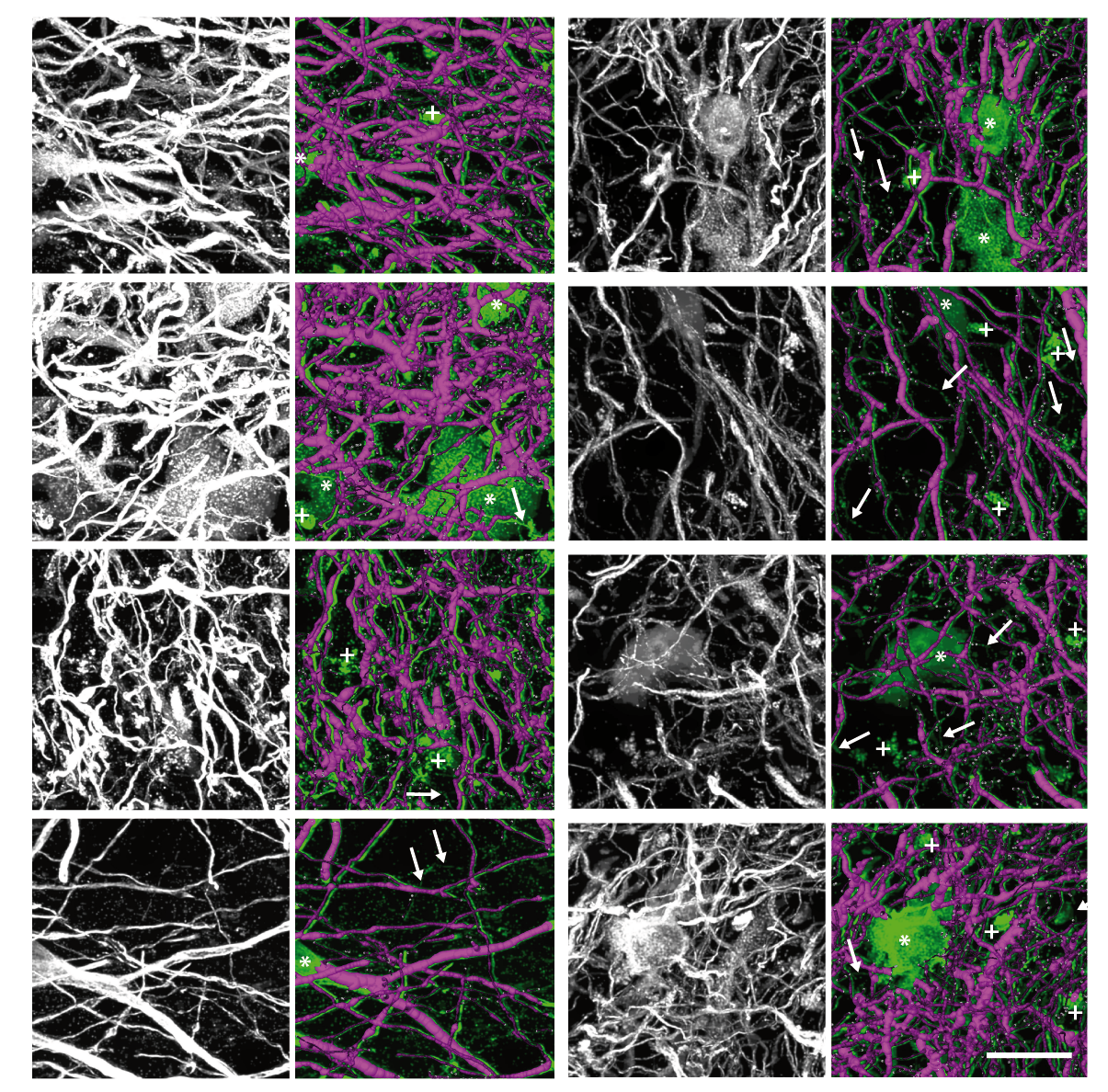


Additional examples of fiber reconstructions from the monkey (MAP2). First two columns on the left are from PIN. Third and fourth column are from the LN/dentate. Scale bar: 25µm.

### Supplementary figure 6:


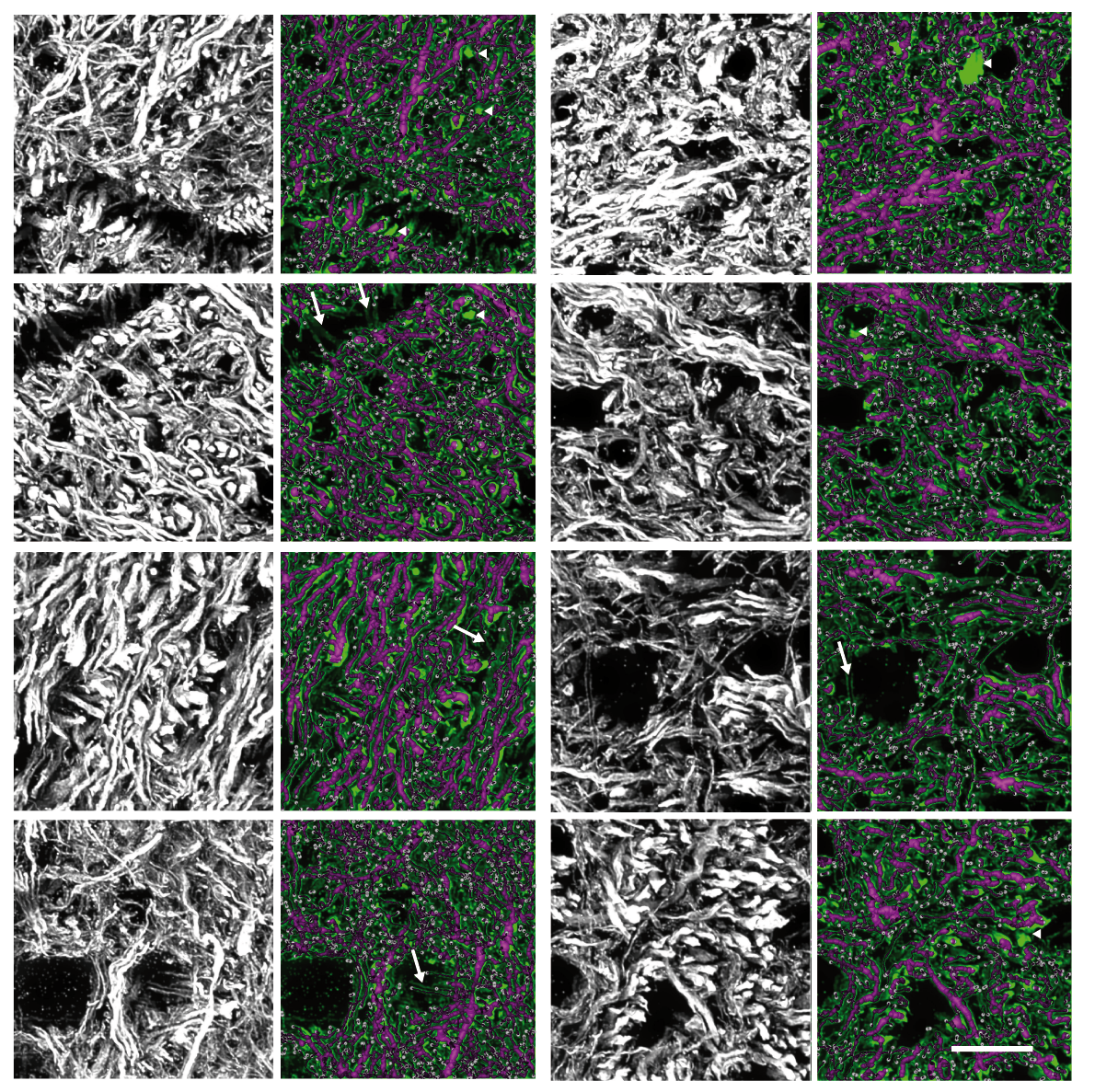


Additional examples of fiber reconstructions from the rat (PCax/PCP2). First two columns on the left are from MN. Third and fourth column are from the AIN. Scale bar: 15µm.

### Supplementary figure 7:


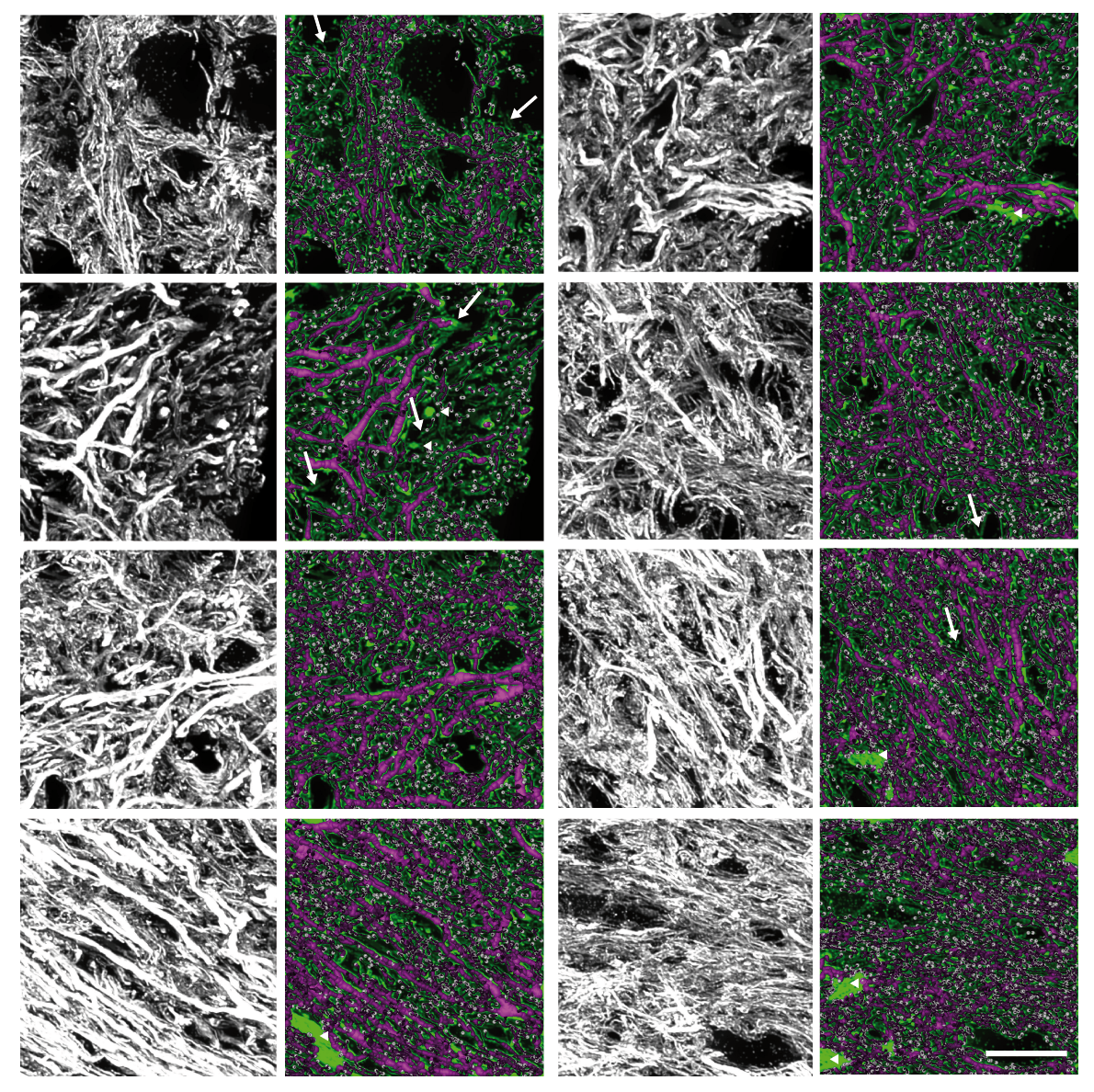


Additional examples of fiber reconstructions from the rat (PCax/PCP2). First two columns on the left are from PIN. Third and fourth column are from the LN/ dentate. Scale bar: 15µm.

### Supplementary figure 8:


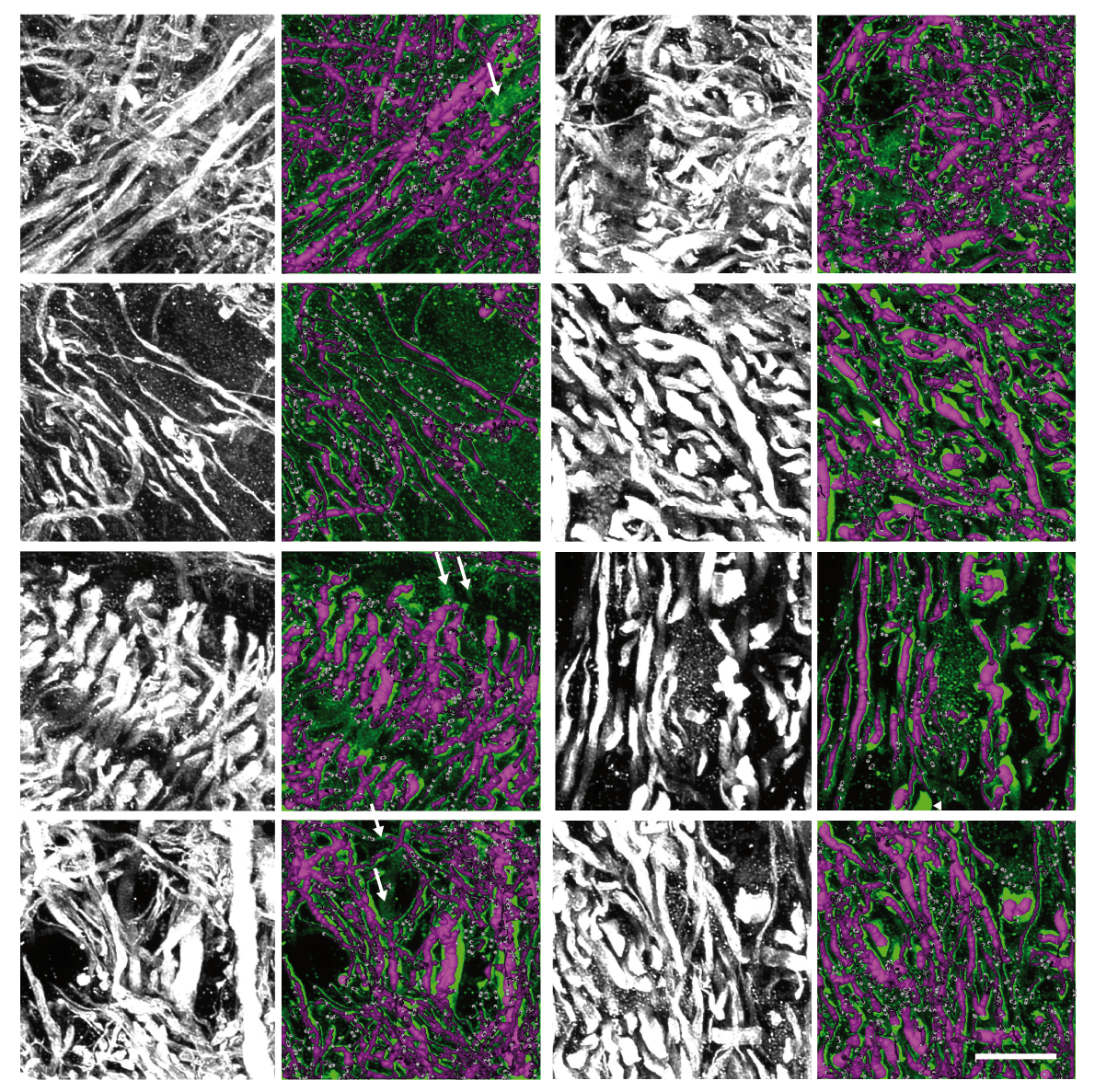


Additional examples of fiber reconstructions from the macaca (PCax/PCP2). First two columns on the left are from MN. Third and fourth column are from the AIN. Scale bar: 15µm.

### Supplementary figure 9:


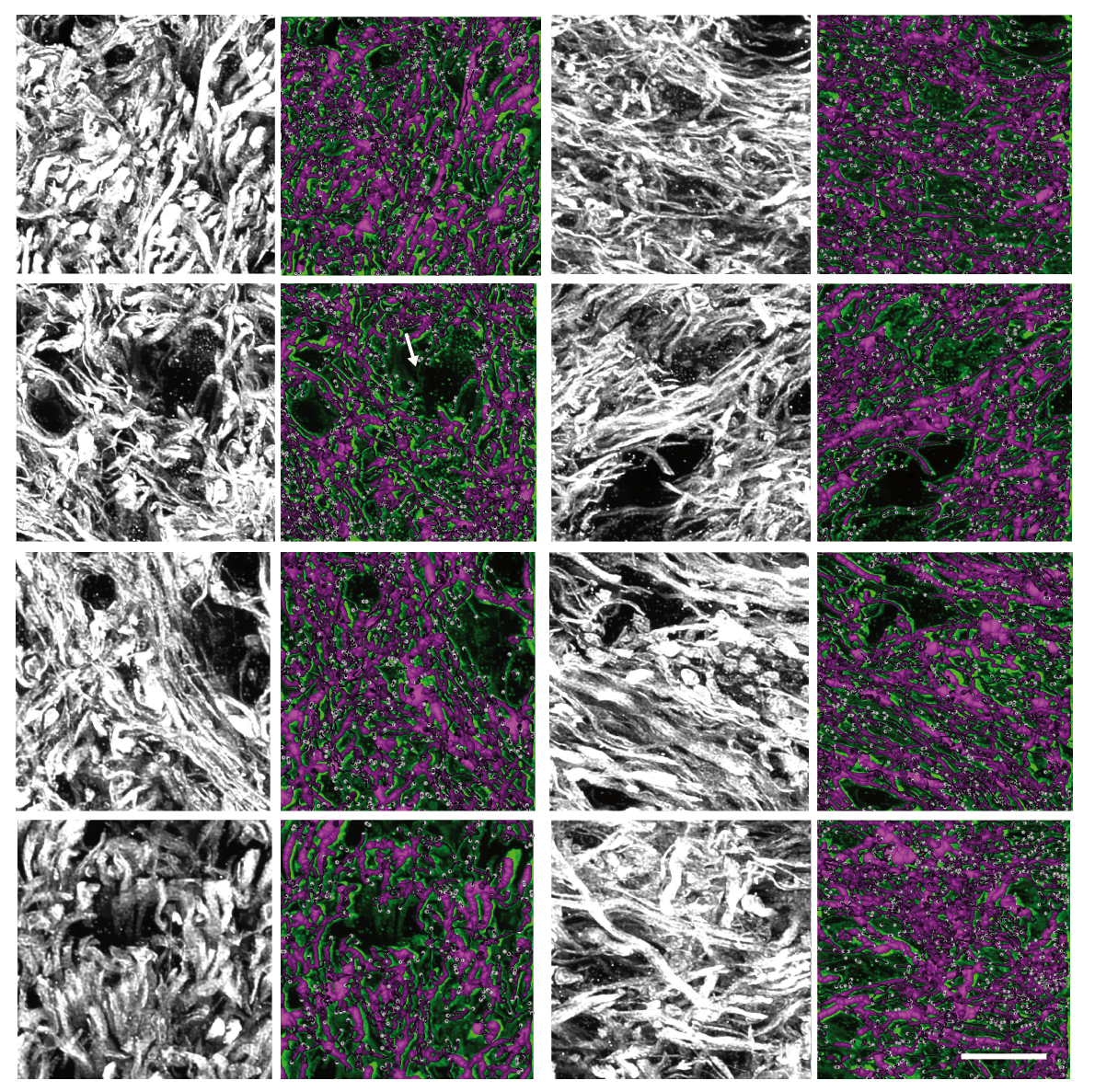


Additional examples of fiber reconstructions from the macaca (PCax/PCP2). First two columns on the left are from PIN. Third and fourth column are from the LN/ dentate. Scale bar: 15µm.

### Supplementary data: Estimating PCax length in rats and rhesus monkey

To obtain the axon length per individual Purkinje cell, we divided the total amount of DCN PCax by the total number of Purkinje cells. The total amount of PCax in rats was obtained by multiplying the length density by the DCN volume: 427m/mm^3 * 6mm^3= 2.56 km. The number of Purkinje cells in the rat cerebellum amounts to 3.6*10^5 ([Harvey and Napper, 1988](#_ENREF_22)) which then yields 7.1mm per Purkinje cell. In the case of the rhesus monkey, we have a PCax density of 400m/mm^3, DCN volume of 188mm^3 and 2.7*10^6 Purkinje cells (counts from this study; CE > 0.05) which yields a length of 27.9mm per Purkinje cell. The Purkinje cell number count of the rhesus monkey is close to previous estimates, with a range of 2.05-3.7*10^6 ([Caddy and Biscoe, 1979](#_ENREF_10)).
